# Digitale Pathologie in der Dermatologie – Status quo, Anwendungen und Perspektiven

**DOI:** 10.1111/ddg.70482

**Published:** 2026-08-04

**Authors:** Cornelia S. L. Müller, Torsten Hansen

**Affiliations:** ^1^ MVZ für Histologie Zytologie und molekulare Diagnostik Trier; ^2^ Universität des Saarlandes Medizinische Fakultät Kirrberger Str. 100, 66421 Homburg/ Saar Deutschland

**Keywords:** Digitale Pathologie, Dermatopathologie, Künstliche Intelligenz, Qualitätssicherung, Telepathologie, Whole‐Slide‐Imaging, Artificial intelligence, Digital pathology, Dermatopathology, Quality assurance, Telepathology, Whole‐slide imaging

## Abstract

Die digitale Pathologie hat sich in den vergangenen Jahren zu einem etablierten Bestandteil der diagnostischen Routine entwickelt. Durch *Whole‐Slide‐Imaging* können histologische Präparate vollständig digitalisiert und am Bildschirm befundet werden. Dies eröffnet neue Möglichkeiten für Diagnostik, Konsile, Archivierung, Lehre und Qualitätssicherung. Insbesondere in der Dermatologie und Dermatopathologie bietet die digitale Pathologie Vorteile bei der Beurteilung entzündlicher Dermatosen, melanozytärer Läsionen und epithelialer Hauttumoren sowie bei immunhistochemischen Zusatzuntersuchungen. Dieser Artikel gibt einen Überblick über die technischen Grundlagen digitaler Pathologiesysteme, beschreibt Arbeitsabläufe und Implementierung im klinischen Alltag und stellt Anforderungen an Qualitätssicherung und Validierung dar. Darüber hinaus werden zentrale Einsatzgebiete in der Dermatologie sowie Vorteile und Limitationen der digitalen Befundung diskutiert. Ein eigenständiger Schwerpunkt liegt auf der Rolle künstlicher Intelligenz als unterstützendes Werkzeug in der dermatopathologischen Diagnostik. Ziel des Artikels ist es, Dermatologen und Pathologen eine fundierte Grundlage für die sachgerechte Bewertung und den klinischen Einsatz digitaler Pathologie zu vermitteln und aktuelle Entwicklungen realistisch einzuordnen.

## EINLEITUNG

Die digitale Pathologie hat sich in den vergangenen Jahren von einer primär technischen Innovation zu einem etablierten Bestandteil der diagnostischen Routine entwickelt. Insbesondere durch die Einführung des *Whole‐Slide‐Imagings* (WSI), bei dem vollständige histologische Präparate digitalisiert und hochauflösend am Bildschirm beurteilt werden können, ergeben sich neue Möglichkeiten für Diagnostik, Konsultation, Lehre und Qualitätssicherung.^1^, ^2^ Parallel dazu hat der Einsatz rechnergestützter Bildanalyse und künstlicher Intelligenz (KI) in der Pathologie erheblich an Bedeutung gewonnen.^3^

In der Dermatologie und Dermatopathologie besitzt die digitale Pathologie ein besonderes Potenzial. Die hohe morphologische Vielfalt entzündlicher Dermatosen, melanozytärer Läsionen sowie epithelialer Hauttumoren stellt hohe Anforderungen an Erfahrung, Vergleichbarkeit und interdisziplinären Austausch. Digitale Präparate ermöglichen eine ortsunabhängige Befundung, erleichtern dermatopathologische Konsile und unterstützen die standardisierte Archivierung histologischer Befunde. Mehrere Studien zur Anwendung von WSI in der Dermatopathologie zeigen eine hohe diagnostische Übereinstimmung zwischen digitaler und konventioneller Glasbefundung bei adäquater technischer Qualität und strukturierter Validierung.^4^, ^5^

Trotz der zunehmenden Verfügbarkeit digitaler Systeme bestehen weiterhin Herausforderungen hinsichtlich Implementierung, *Workflow*‐Integration und rechtlicher Rahmenbedingungen. Die Umstellung von der mikroskopischen Befundung auf eine primär digitale Diagnostik erfordert sorgfältige Planung unter Berücksichtigung von Scannertechnik, Bildqualität, IT‐Infrastruktur, Datenspeicherung und Datenschutz. Internationale Erfahrungen zeigen, dass die erfolgreiche Einführung digitaler Pathologiesysteme maßgeblich von standardisierten Validierungsprozessen und schrittweiser Integration in den Routinelaborbetrieb abhängt.^6^, ^7^

Ein weiterer Schwerpunkt der aktuellen Entwicklung ist der Einsatz KI‐basierter Assistenzsysteme. Diese Anwendungen werden insbesondere zur Detektion, Klassifikation und Quantifizierung histologischer Strukturen eingesetzt und können die ärztliche Befundung unterstützen. Systematische Übersichtsarbeiten und Metaanalysen belegen, dass KI‐gestützte Verfahren in ausgewählten Anwendungsbereichen eine hohe diagnostische Leistungsfähigkeit erreichen können, gleichzeitig jedoch Limitationen hinsichtlich Generalisierbarkeit, Datenqualität und klinischer Validierung bestehen.^8^, ^9^ Der Einsatz von KI erfolgt derzeit ergänzend zur ärztlichen Diagnostik und ersetzt diese nicht.^3^

Ziel dieses CME‐Artikels ist es, einen strukturierten Überblick über die Grundlagen der digitalen Pathologie zu geben, relevante Einsatzgebiete in der Dermatologie darzustellen und Chancen sowie Limitationen realistisch einzuordnen. Neben technischen und organisatorischen Aspekten werden auch Qualitätssicherung, Validierungsstrategien und zukünftige Perspektiven berücksichtigt. Der Beitrag soll Dermatologen und Pathologen eine fundierte Grundlage für die Bewertung und den sachgerechten Einsatz digitaler Pathologiesysteme in der klinischen Praxis vermitteln.

Digitale Pathologie bezeichnet die Digitalisierung vollständiger histologischer Präparate zur primär digitalen Befundung am Bildschirm.

## TECHNISCHE GRUNDLAGEN DER DIGITALEN PATHOLOGIE

**ABBILDUNG 1:**
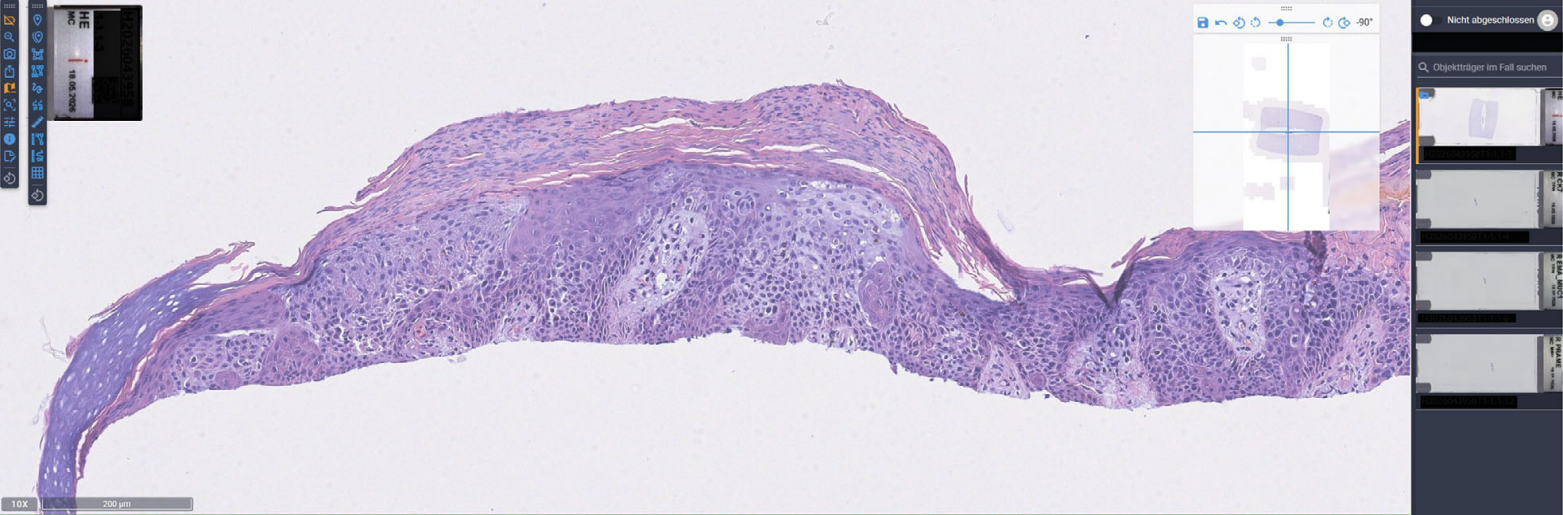
Beispiel eines digitalisierten histologischen Präparats (Whole‐Slide‐Image) in einer digitalen Viewer‐Umgebung.

Die digitale Pathologie basiert auf der vollständigen Digitalisierung histologischer Präparate durch das sogenannte *Whole‐Slide‐Imaging* (WSI). Dabei werden konventionelle Glasobjektträger mit speziellen Scannern hochauflösend erfasst und als digitale Gesamtbilder gespeichert, die eine mikroskopische Beurteilung am Bildschirm ermöglichen^1^, ^2^ (Abbildung 1). Im Gegensatz zu statischen Einzelaufnahmen erlaubt WSI das kontinuierliche Navigieren durch das gesamte Präparat bei unterschiedlichen Vergrößerungsstufen und bildet damit die Grundlage für eine primär digitale Befundung.

### Whole‐Slide‐Imaging und Scannertechnologie

WSI‐Scanner erfassen histologische Schnitte typischerweise bei einer optischen Vergrößerung von 20× oder 40×, was einer effektiven Auflösung im Submikrometerbereich entspricht. Moderne Systeme verfügen über automatisierte Fokus‐ und Belichtungsalgorithmen, um eine gleichbleibende Bildqualität über das gesamte Präparat zu gewährleisten. Abhängig vom Scanner können sowohl Hellfeldpräparate als auch immunhistochemische Färbungen und Spezialfärbungen digitalisiert werden.^1^ Die Scanzeit variiert je nach Präparatgröße, Auflösung und Gerätemodell und liegt in der Regel zwischen wenigen Minuten und über zehn Minuten pro Objektträger. Moderne Scanner ermöglichen zudem das automatisierte sequentielle Scannen größerer Probenserien, sodass dutzende bis hunderte Objektträger ohne manuellen Eingriff verarbeitet werden können, beispielsweise im kontinuierlichen Betrieb oder über Nacht.

Die entstehenden Bilddateien weisen aufgrund der hohen Auflösung ein großes Datenvolumen auf. Unkomprimierte WSI‐Dateien können mehrere Gigabyte umfassen, weshalb in der Praxis meist verlustarme oder verlustbehaftete Kompressionsverfahren eingesetzt werden. Unterschiedliche proprietäre und offene Dateiformate sind im Einsatz, was die standardisierte Archivierung und systemübergreifende Nutzung weiterhin erschwert.^2^

### *Viewer*‐Systeme und digitale Befundung

Zur Betrachtung der digitalisierten Präparate werden spezielle *Viewer*‐Softwarelösungen eingesetzt. Diese ermöglichen das stufenlose Vergrößern, Verschieben und Markieren von Befundarealen sowie den Vergleich mehrerer Schnitte oder Färbungen parallel auf dem Bildschirm. Je nach System können *Viewer* lokal installiert oder webbasiert betrieben werden. Für die diagnostische Nutzung ist eine farbgetreue Darstellung essenziell, weshalb die Kalibrierung der Monitore und eine ausreichende Bildschirmauflösung eine zentrale Rolle spielen.^6^ Die digitale Befundung erfordert zudem die Anpassung ergonomischer Aspekte. Studien zeigen, dass Bildschirmgröße, Auflösung und Arbeitsplatzgestaltung Einfluss auf Befunddauer und Ermüdung haben können, insbesondere bei komplexen dermatopathologischen Fragestellungen mit hoher morphologischer Detaildichte.^4^

### IT‐Infrastruktur und Datenspeicherung

Eine leistungsfähige IT‐Infrastruktur ist Voraussetzung für den routinemäßigen Einsatz digitaler Pathologiesysteme. Neben ausreichender Rechenleistung sind vor allem Speicherlösungen mit hoher Kapazität und Datensicherheit erforderlich. Je nach Laborgröße entstehen jährlich mehrere Terabyte an Bilddaten. Die Speicherung kann lokal, serverbasiert oder in hybriden Systemen erfolgen. Cloudbasierte Lösungen werden zunehmend diskutiert, erfordern jedoch besondere datenschutzrechtliche und organisatorische Maßnahmen.^7^

Der Zugriff auf digitale Präparate erfolgt über gesicherte Netzwerke und Benutzerverwaltungssysteme. Für Konsile und Zweitmeinungen können zeitlich begrenzte Zugriffsrechte vergeben werden, was den interdisziplinären Austausch erleichtert und wodurch physische Transportwege in vielen Fällen entfallen und durch digitale Datenübertragung ersetzt werden.^5^

### Integration von Bildanalyse und KI

Digitale Präparate bilden die Grundlage für computergestützte Bildanalyse und KI‐basierte Assistenzsysteme. Diese Anwendungen greifen direkt auf die WSI‐Daten zu und analysieren definierte Bildparameter wie Zellzahl, Färbeintensität oder morphologische Muster. Technisch erfordern solche Systeme standardisierte Bildqualität und konsistente Färbeprotokolle, da Abweichungen die Analyseergebnisse beeinflussen können.^3^, ^9^

Der Einsatz dieser Technologien erfolgt derzeit überwiegend ergänzend zur ärztlichen Befundung. Eine vollständige Automatisierung diagnostischer Entscheidungen ist nicht vorgesehen, vielmehr stehen Unterstützung, Quantifizierung und Reproduzierbarkeit im Vordergrund.^8^

KI‐basierte Assistenzsysteme können die dermatopathologische Diagnostik unterstützen, ersetzen jedoch nicht die ärztliche Befundung.

### Zusammenfassung der technischen Voraussetzungen

Die erfolgreiche Implementierung digitaler Pathologiesysteme setzt ein Zusammenspiel aus Scannertechnologie, *Viewer*‐Software, IT‐Infrastruktur und standardisierten Arbeitsabläufen voraus. Insbesondere in der Dermatopathologie ist eine hohe Bildqualität essenziell, um die feinen morphologischen Details zuverlässig beurteilen zu können. Eine sorgfältige technische Planung bildet daher die Grundlage für eine sichere und effiziente digitale Diagnostik.

Das *Whole‐Slide‐Imaging* ermöglicht die hochauflösende Erfassung kompletter histologischer Schnitte und bildet die technische Grundlage der digitalen Pathologie.

Eine diagnostisch sichere digitale Befundung setzt eine konstante Bildqualität, geeignete Viewer‐Systeme und eine leistungsfähige IT‐Infrastruktur voraus.

### WORKFLOW UND IMPLEMENTIERUNG

Die Implementierung digitaler Pathologiesysteme in den diagnostischen Routinebetrieb erfordert eine Anpassung bestehender Arbeitsabläufe sowie eine enge Abstimmung zwischen Pathologie, IT‐Abteilung und klinischen Partnern. Ziel ist es, digitale Prozesse nahtlos in den etablierten histologischen Arbeitsablauf zu integrieren, ohne die diagnostische Qualität oder Effizienz zu beeinträchtigen.^7^, ^8^

Der digitale Workflow beginnt in der Regel nach der konventionellen histologischen Aufarbeitung. Nach Schneiden, Färben und Eindecken der Präparate werden die Glasobjektträger dem WSI‐Scanner zugeführt. Die digitalisierten Schnitte stehen anschließend über das *Viewer*‐System zur Befundung zur Verfügung. Je nach Laborstruktur kann die digitale Befundung parallel zur Glasbefundung erfolgen oder diese schrittweise ersetzen. In vielen Einrichtungen hat sich eine Übergangsphase mit hybriden Workflows etabliert, um die diagnostische Sicherheit zu gewährleisten und die Akzeptanz zu fördern.^1^

Ein zentraler Aspekt der Implementierung ist die Auswahl geeigneter Scanner‐ und Softwaresysteme. Neben Bildqualität und Scanleistung spielen Aspekte wie Benutzerfreundlichkeit, Schnittstellen zu Laborinformationssystemen (LIS) und langfristige Wartung eine entscheidende Rolle. Die Integration digitaler Präparate in bestehende LIS‐Strukturen ermöglicht eine direkte Verknüpfung von Bilddaten, Befunden und klinischen Informationen und unterstützt eine effiziente Fallbearbeitung.^2^

Die Befundung am Bildschirm unterscheidet sich in einigen Punkten von der konventionellen Mikroskopie. Untersuchungen zeigen, dass sich Befundzeiten und Navigationsstrategien initial verändern, sich jedoch mit zunehmender Erfahrung stabilisieren. Schulungen und standardisierte Einführungsprogramme sind daher essenziell, um eine sichere und effiziente Nutzung digitaler Systeme zu gewährleisten.^4^ Digitale Pathologiesysteme erleichtern zudem dermatopathologische Konsile und Zweitmeinungen. Durch den gesicherten Zugriff auf digitale Präparate können externe Experten zeitnah eingebunden werden, ohne dass physische Objektträger versendet werden müssen. Dies verkürzt Bearbeitungszeiten und reduziert das Risiko von Präparatverlusten oder ‐beschädigungen.^5^

Ein zusätzlicher Implementierungsaspekt betrifft die Archivierung und Langzeitverfügbarkeit digitaler Präparate. Digitale Archive ermöglichen den schnellen Zugriff auf Vorbefunde und unterstützen Verlaufskontrollen, insbesondere bei chronischen entzündlichen Dermatosen oder rezidivierenden Hauttumoren. Gleichzeitig erfordern sie klare Regelungen zur Speicherstrategie, Datensicherung und Zugriffsberechtigung.^7^

Ein weiterer relevanter Aspekt ist die Interoperabilität digitaler Pathologiesysteme. Unterschiedliche Anbieter nutzen teilweise proprietäre Datenformate und Softwarelösungen, was den Austausch zwischen Systemen sowie einen späteren Anbieterwechsel erschweren kann. Dies kann zu einer sogenannten *Vendor‐Lock‐in*‐Situation führen, bei der eine Abhängigkeit von einem einzelnen Anbieter entsteht und ein Systemwechsel nur mit erheblichem technischem und organisatorischem Aufwand möglich ist. Offene Standards und kompatible Schnittstellen sind daher entscheidend, um langfristige Datenverfügbarkeit und eine flexible Systemintegration zu gewährleisten.

Die schrittweise Einführung digitaler Pathologie hat sich als praktikabler Ansatz erwiesen. Pilotprojekte mit ausgewählten Fallgruppen oder Subdisziplinen erlauben eine frühzeitige Identifikation technischer und organisatorischer Herausforderungen. Auf dieser Basis können Arbeitsabläufe angepasst und standardisiert werden, bevor eine vollständige Umstellung erfolgt.^6^

Zusammenfassend stellt die Implementierung digitaler Pathologie einen multidisziplinären Prozess dar, der technische, organisatorische und personelle Aspekte gleichermaßen berücksichtigt. Eine strukturierte Planung und kontinuierliche Evaluation sind entscheidend für eine erfolgreiche Integration in den klinischen Alltag.

Die Einführung digitaler Pathologie erfordert eine strukturierte Anpassung bestehender Arbeitsabläufe und eine enge Abstimmung zwischen Pathologie und IT.

Hybride Arbeitsabläufe mit paralleler Glas‐ und Digitalbefundung haben sich als praktikabler Übergang in der Implementierungsphase etabliert.

## QUALITÄTSSICHERUNG UND VALIDIERUNG

Die Einführung digitaler Pathologiesysteme in die diagnostische Routine erfordert eine strukturierte Qualitätssicherung und eine formale Validierung, um eine gleichwertige diagnostische Leistung im Vergleich zur konventionellen Glasbefundung sicherzustellen. Internationale Empfehlungen betonen, dass digitale Systeme vor dem Routineeinsatz unter realen Arbeitsbedingungen geprüft werden müssen.^1^, ^6^

Die Validierung umfasst in der Regel den Vergleich digitaler Präparate mit den entsprechenden Glasobjektträgern über ein repräsentatives Fallspektrum hinweg. Dabei sollten unterschiedliche diagnostische Kategorien, Färbungen und Schwierigkeitsgrade berücksichtigt werden. Ziel ist es, die diagnostische Übereinstimmung sowie potenzielle systematische Abweichungen zu erfassen. Studien zeigen, dass bei adäquater Bildqualität und strukturierter Durchführung eine hohe Übereinstimmung zwischen digitaler und konventioneller Befundung erreicht werden kann.^3^, ^5^

Ein wesentlicher Bestandteil der Qualitätssicherung ist die kontinuierliche Überwachung technischer Parameter. Dazu zählen Scannerleistung, Fokusgenauigkeit, Farbwiedergabe und Monitorqualität. Regelmäßige Wartung der Geräte sowie die Kalibrierung der Bildschirme sind notwendig, um eine konstante Bildqualität zu gewährleisten. Veränderungen in Färbeprotokollen oder Schnittqualität können sich unmittelbar auf die digitale Darstellung auswirken und sollten daher in bestehende Qualitätssicherungsmaßnahmen integriert werden.^2^

Auch organisatorische Aspekte spielen eine zentrale Rolle. Klare Regelungen zur Befundfreigabe, Dokumentation und Archivierung sind erforderlich, um die Nachvollziehbarkeit diagnostischer Entscheidungen sicherzustellen. Der digitale Zugriff auf Präparate muss über gesicherte Benutzerverwaltungssysteme erfolgen, insbesondere bei Konsilen und externen Zweitmeinungen.^7^

Für den Einsatz KI‐basierter Assistenzsysteme gelten zusätzliche Anforderungen. Diese betreffen insbesondere die klinische Validierung, die regelmäßige Leistungsüberprüfung sowie die transparente Darstellung der Anwendungsgrenzen. Der Einsatz solcher Systeme sollte stets unter ärztlicher Aufsicht erfolgen und klar von der eigenständigen diagnostischen Entscheidung abgegrenzt sein.^3^, ^8^

Insgesamt bildet eine strukturierte Qualitätssicherung die Grundlage für eine sichere und zuverlässige Nutzung digitaler Pathologiesysteme im klinischen Alltag.

Digitale Pathologiesysteme müssen vor dem Routineeinsatz unter realen Arbeitsbedingungen validiert werden, um eine diagnostische Gleichwertigkeit zur Glasbefundung sicherzustellen.

## ORDER ENTRY UND DIGITALE ANFORDERUNG

Die digitale Pathologie setzt zunehmend bereits bei der Anforderung histopathologischer Untersuchungen an. Elektronische *Order‐Entry*‐Systeme ermöglichen die strukturierte Erfassung klinischer Fragestellungen, Entnahmelokalisationen und relevanter Zusatzinformationen bereits vor der histologischen Aufarbeitung. Diese Informationen stehen der Pathologie unmittelbar digital zur Verfügung und können direkt in den weiteren diagnostischen Arbeitsablauf integriert werden.

Durch eine standardisierte digitale Anforderung lassen sich Medienbrüche reduzieren und die Nachvollziehbarkeit diagnostischer Prozesse verbessern. Insbesondere in der Dermatologie, in der multiple Biopsien und serielle Entnahmen häufig sind, kann eine strukturierte *Order‐Entry*‐Lösung zur Übersichtlichkeit und Zuordnung von Präparaten beitragen. Die Verknüpfung von Order Entry, Laborinformationssystem und digitalen Präparaten unterstützt zudem eine konsistente Dokumentation.

Die Implementierung digitaler *Order‐Entry*‐Systeme erfordert eine enge Abstimmung zwischen einsendenden Einrichtungen und Pathologie. Einheitliche Datenformate, klare Verantwortlichkeiten und eine Anpassung bestehender Abläufe sind Voraussetzung für einen reibungslosen Einsatz. Der diagnostische Nutzen ergibt sich insbesondere aus der verbesserten Informationsverfügbarkeit, nicht aus einer Automatisierung der Befundentscheidung.

Die Einführung digitaler *Order‐Entry*‐Systeme ist jedoch mit Herausforderungen verbunden, insbesondere durch uneinheitliche Schnittstellen, unvollständige klinische Angaben und eine variable Akzeptanz in der täglichen Praxis.

Digitale *Order‐Entry*‐Systeme können den pathologischen Workflow unterstützen, sind jedoch von der Qualität klinischer Angaben, kompatiblen Schnittstellen und der Akzeptanz im klinischen Alltag abhängig.

## RECHTLICHE, HAFTUNGSRECHTLICHE UND ABRECHNUNGSTECHNISCHE ASPEKTE

Die Einführung digitaler Pathologiesysteme in die klinische Routine ist nicht nur mit technischen und organisatorischen Anforderungen verbunden, sondern wirft auch rechtliche, haftungsrechtliche und abrechnungstechnische Fragestellungen auf. Diese Rahmenbedingungen sind derzeit nicht in allen Bereichen abschließend geklärt und unterliegen einer fortlaufenden Entwicklung.

Aus rechtlicher Sicht ist insbesondere die Verantwortlichkeit für die Befundung digitaler Präparate von Bedeutung. Auch bei Einsatz digitaler Systeme und KI‐basierter Assistenzwerkzeuge verbleibt die diagnostische Verantwortung bei der ärztlichen Person, die den Befund freigibt. Der Einsatz unterstützender Software ändert nichts an der ärztlichen Haftung für diagnostische Entscheidungen. Klare interne Regelungen zur Befundfreigabe und Dokumentation sind daher erforderlich.

Haftungsrechtlich besteht derzeit noch keine einheitliche Rechtsprechung zur Nutzung digitaler Pathologiesysteme und KI‐basierter Anwendungen in der Routinediagnostik. Dies betrifft insbesondere Fragen der Mitverantwortung bei softwaregestützten Analysen sowie den Umgang mit technischen Fehlfunktionen. Eine sorgfältige Validierung, Dokumentation und Qualitätssicherung sind daher von zentraler Bedeutung, um potenzielle Risiken zu minimieren.

Auch abrechnungstechnische Aspekte sind bislang nicht abschließend geregelt. Die Nutzung digitaler Pathologiesysteme und KI‐gestützter Analysen ist derzeit nicht flächendeckend in bestehenden Abrechnungssystemen abgebildet. Dies betrifft sowohl die Vergütung der digitalen Befundung als auch zusätzliche Leistungen im Rahmen digitaler Konsile oder computergestützter Auswertungen. Die konkrete Abrechenbarkeit kann daher je nach Versorgungsbereich und regionalen Regelungen variieren.

Zusammenfassend stellen rechtliche, haftungsrechtliche und abrechnungstechnische Fragestellungen einen wesentlichen Rahmenfaktor bei der Einführung digitaler Pathologie dar. Diese Aspekte sollten bei der Implementierung berücksichtigt und kontinuierlich an aktuelle rechtliche Entwicklungen angepasst werden.

Rechtliche, haftungsrechtliche und abrechnungstechnische Rahmenbedingungen der digitalen Pathologie sind derzeit nicht vollständig geklärt und müssen bei der Implementierung berücksichtigt werden.

## KÜNSTLICHE INTELLIGENZ IN DER DIGITALEN PATHOLOGIE

KI stellt eine wichtige Erweiterung digitaler Pathologiesysteme dar und basiert auf der computergestützten Analyse digitalisierter histologischer Präparate. Die Grundlage bilden Whole‐Slide‐Images, die mithilfe maschineller Lernverfahren ausgewertet werden. Ziel dieser Anwendungen ist es, diagnostische Prozesse zu unterstützen, quantitative Analysen zu ermöglichen und die Reproduzierbarkeit histopathologischer Befunde zu erhöhen.^3^, ^8^

In der dermatopathologischen Diagnostik kommen KI‐basierte Systeme derzeit vor allem als Assistenzwerkzeuge zum Einsatz. Typische Anwendungsbereiche sind die Detektion verdächtiger Areale, die Klassifikation histologischer Muster sowie die Quantifizierung immunhistochemischer Marker. Insbesondere bei zeitintensiven Auswertungen kann dies zur Unterstützung der ärztlichen Befundung beitragen. Voraussetzung hierfür ist eine standardisierte Bildqualität, da Variationen in Färbung, Schnittdicke oder Scanparametern die Analyseergebnisse beeinflussen können.^8^, ^9^

Mehrere Studien zeigen, dass KI‐gestützte Verfahren in ausgewählten Anwendungsbereichen eine hohe diagnostische Leistungsfähigkeit erreichen können. Gleichzeitig bestehen Limitationen hinsichtlich der Übertragbarkeit auf unterschiedliche Laborbedingungen und Patientenkollektive. Die Leistungsfähigkeit solcher Systeme ist maßgeblich von den zugrunde liegenden Trainingsdaten abhängig, was eine sorgfältige klinische Validierung erforderlich macht.^8^ Für den Einsatz in der klinischen Routine sind regulatorische und rechtliche Aspekte von Bedeutung. KI‐basierte Anwendungen unterliegen medizinprodukterechtlichen Anforderungen und müssen entsprechend zugelassen und überwacht werden. Darüber hinaus ist eine klare Abgrenzung zwischen unterstützender Analyse und ärztlicher Entscheidungsfindung erforderlich. Der Einsatz von KI erfolgt daher derzeit ergänzend zur ärztlichen Diagnostik und ersetzt diese nicht.^3^ Für die klinische Akzeptanz KI‐basierter Verfahren ist neben der diagnostischen Leistungsfähigkeit auch die Nachvollziehbarkeit und transparente Darstellung der algorithmischen Ergebnisse von zentraler Bedeutung. Ein weiterer Aspekt betrifft die Integration von KI‐Systemen in bestehende digitale Arbeitsabläufe. Technisch müssen KI‐Anwendungen nahtlos in Viewer‐ und Laborinformationssysteme eingebunden werden, um einen effizienten Einsatz zu ermöglichen. Organisatorisch erfordert dies klare Verantwortlichkeiten, insbesondere im Hinblick auf Ergebnisinterpretation und Befundfreigabe.^6^ Zukünftig könnten KI‐basierte Systeme zur weiteren Standardisierung dermatopathologischer Befunde beitragen und quantitative Auswertungen in größerem Umfang ermöglichen. Derzeit bleibt jedoch eine kritische Bewertung der Einsatzmöglichkeiten und Limitationen essenziell, um einen sachgerechten Einsatz in der klinischen Praxis sicherzustellen.^2^

KI‐basierte Anwendungen in der digitalen Pathologie dienen der unterstützenden Analyse digitaler Präparate und erfordern eine sorgfältige klinische Validierung sowie ärztliche Kontrolle.

## EINSATZGEBIETE DER DIGITALEN PATHOLOGIE IN DER DERMATOLOGIE

Die digitale Pathologie findet in der Dermatologie und Dermatopathologie in unterschiedlichen diagnostischen Kontexten Anwendung. Durch die vollständige Digitalisierung histologischer Präparate können dermatologische Fragestellungen effizient bearbeitet, Befunde vergleichbar dokumentiert und interdisziplinäre Prozesse unterstützt werden. Die Einsatzgebiete reichen von entzündlichen Dermatosen über pigmentierte Läsionen bis hin zu epithelialen Hauttumoren und immunhistochemischen Untersuchungen,^2^, ^5^ (Tabelle 1).

**TABELLE 1:** Zentrale Einsatzgebiete der digitalen Pathologie in der Dermatologie und ihr klinischer Nutzen.

Einsatzgebiet	Nutzen der digitalen Pathologie
Entzündliche Dermatosen	Vergleich digitaler Archive, Verlaufskontrollen, standardisierte Befundung
Melanozytäre Läsionen	Digitale Konsile, parallele Betrachtung serieller Schnitte und IHC
Epitheliale Hauttumoren	Beurteilung von Tumorarchitektur, Resektionsrändern und Rezidiven
Immunhistochemie	Parallele Markeransicht, unterstützende Quantifizierung
Konsile und Zweitmeinungen	Ortsunabhängiger Zugriff, zeitnahe fachliche Rückmeldung
Lehre und Weiterbildung	Digitale Fallarchive, strukturierte Wissensvermittlung

### Entzündliche Dermatosen

Bei entzündlichen Hauterkrankungen spielt die Beurteilung subtiler histomorphologischer Veränderungen eine zentrale Rolle. Digitale Präparate ermöglichen eine strukturierte Befundung und den Vergleich aktueller Befunde mit Vorpräparaten desselben Patienten. Insbesondere bei chronisch‐rezidivierenden Dermatosen kann der Zugriff auf digitale Archive die Verlaufskontrolle erleichtern. Darüber hinaus unterstützen digitale Systeme die standardisierte Dokumentation typischer histologischer Muster und fördern die konsistente Befundung innerhalb größerer Einrichtungen.^4^

### Melanozytäre Läsionen

Die Diagnostik melanozytärer Läsionen stellt hohe Anforderungen an die morphologische Detailerkennung und die Beurteilung architektonischer Kriterien. Digitale Pathologie ermöglicht die gleichzeitige Betrachtung mehrerer Schnittebenen und immunhistochemischer Zusatzfärbungen auf einem Bildschirm. Studien zeigen, dass bei adäquater Bildqualität eine vergleichbare diagnostische Sicherheit zwischen digitaler und konventioneller Befundung erreicht werden kann.^1^ Digitale Konsile sind insbesondere bei diagnostisch schwierigen Läsionen von Bedeutung und unterstützen den fachlichen Austausch zwischen spezialisierten Zentren.

### Epitheliale Hauttumoren

Bei epithelialen Hauttumoren wie Basalzell‐ und Plattenepithelkarzinomen ermöglicht die digitale Pathologie eine effiziente Beurteilung von Tumorarchitektur, Infiltrationstiefe und Resektionsrändern. Die digitale Archivierung erleichtert zudem den Vergleich von Primärtumoren und Rezidiven. In der operativen Dermatologie kann der zeitnahe digitale Zugriff auf Befunde zur Therapieplanung beitragen, insbesondere bei komplexen oder multiplen Läsionen.^5^

### Immunhistochemie und Zusatzuntersuchungen

Digitale Systeme bieten Vorteile bei der Beurteilung immunhistochemischer Färbungen. Bei fluoreszenzbasierten Färbungen besteht darüber hinaus der Vorteil, dass digitale Präparate im Gegensatz zu physischem Material nicht ausbleichen. Durch standardisierte Darstellungsoptionen und die Möglichkeit der parallelen Betrachtung mehrerer Marker kann die Interpretation erleichtert werden. Darüber hinaus bilden digitale Präparate die Grundlage für rechnergestützte Quantifizierungen, etwa bei Proliferationsmarkern oder Expressionsanalysen. Voraussetzung hierfür ist eine konsistente Färbequalität und eine validierte technische Umgebung.^3^, ^9^

### Konsile, Zweitmeinungen und Vernetzung

Ein wesentlicher Einsatzbereich der digitalen Pathologie ist die Durchführung dermatopathologischer Konsile. Digitale Präparate können ortsunabhängig und zeitnah zur Verfügung gestellt werden, wodurch physische Transportwege entfallen und durch digitale Datenübertragung ersetzt werden, die in der Regel deutlich kürzere Übertragungszeiten ermöglicht. Dies fördert die Vernetzung zwischen dermatologischen Praxen, Kliniken und spezialisierten Zentren und trägt zur Qualitätssicherung bei, insbesondere bei seltenen oder komplexen Erkrankungen.^7^

### Lehre und Weiterbildung

Neben der Routinediagnostik spielt die digitale Pathologie eine zunehmende Rolle in der Aus‐ und Weiterbildung. Digitale Fallarchive ermöglichen den strukturierten Zugang zu typischen und seltenen Befunden und unterstützen die standardisierte Vermittlung dermatopathologischer Inhalte. Dieser Aspekt gewinnt insbesondere vor dem Hintergrund begrenzter Verfügbarkeit von Glaspräparaten an Bedeutung.^6^

Zusammenfassend bietet die digitale Pathologie vielfältige Einsatzmöglichkeiten in der Dermatologie, die diagnostische Prozesse unterstützen und den interdisziplinären Austausch fördern.

In der Dermatopathologie unterstützt die digitale Pathologie insbesondere Konsile, Verlaufskontrollen und die standardisierte Befunddokumentation.

Die digitale Beurteilung melanozytärer Läsionen erfordert eine hohe Bildqualität und eine sorgfältige Qualitätssicherung aufgrund der feinen morphologischen Details.

## VORTEILE UND LIMITATIONEN DER DIGITALEN PATHOLOGIE

Die digitale Pathologie bietet eine Reihe von Vorteilen für die dermatologische Diagnostik, ist jedoch auch mit technischen, organisatorischen und strukturellen Limitationen verbunden. Eine realistische Bewertung beider Aspekte ist Voraussetzung für einen sachgerechten Einsatz im klinischen Alltag.^1^, ^2^

### Vorteile

Ein wesentlicher Vorteil der digitalen Pathologie liegt in der ortsunabhängigen Verfügbarkeit histologischer Präparate. Digitale Objektträger können zeit‐ und ortsunabhängig befundet werden, was insbesondere für dermatopathologische Konsile und Zweitmeinungen von Bedeutung ist. Dadurch lassen sich Transportzeiten physischer Präparate vermeiden und Bearbeitungszeiten verkürzen.^5^, ^7^

Die digitale Archivierung histologischer Präparate ermöglicht einen schnellen Zugriff auf Vorbefunde und erleichtert Verlaufskontrollen. Dies ist insbesondere bei chronischen entzündlichen Dermatosen, melanozytären Läsionen und rezidivierenden Hauttumoren relevant. Darüber hinaus unterstützt die digitale Dokumentation eine standardisierte Befundung und trägt zur Vergleichbarkeit diagnostischer Entscheidungen bei.^4^

Digitale Präparate bilden zudem die Grundlage für computergestützte Bildanalysen und KI‐basierte Assistenzsysteme. Diese können quantitative Auswertungen unterstützen und zur Reproduzierbarkeit diagnostischer Parameter beitragen. In ausgewählten Anwendungsbereichen konnte eine hohe diagnostische Leistungsfähigkeit solcher Systeme gezeigt werden, sofern eine adäquate technische und klinische Validierung vorliegt.^3^, ^8^

Ein weiterer Vorteil besteht in der Nutzung digitaler Pathologie für Lehre und Weiterbildung. Digitale Fallarchive ermöglichen einen strukturierten Zugang zu typischen und seltenen dermatopathologischen Befunden und unterstützen die standardisierte Wissensvermittlung unabhängig von der Verfügbarkeit physischer Präparate.^6^

### Limitationen

Zu den wesentlichen Limitationen zählen die hohen Anforderungen an technische Infrastruktur und Datenspeicherung. Die Digitalisierung hochauflösender Präparate führt zu erheblichen Datenmengen, die leistungsfähige Speicherlösungen und stabile Netzwerkinfrastrukturen erfordern. Dies kann insbesondere für kleinere Einrichtungen eine Herausforderung darstellen.^2^

Technische Faktoren wie Scanartefakte, Fokusprobleme oder unzureichende Farbwiedergabe können die diagnostische Beurteilbarkeit beeinträchtigen.

Ein weiterer limitierender Aspekt betrifft die fehlende Möglichkeit zur Polarisationsmikroskopie bei digitalisierten Präparaten. Bestimmte diagnostische Fragestellungen, wie der Nachweis von Amyloid (Kongorot) oder die Beurteilung von Fremdmaterial, können dadurch erschwert sein. Während experimentelle Ansätze zur polarisationsempfindlichen digitalen Bildgebung existieren, ist eine entsprechende Funktion in gängigen Routine‐Scannern derzeit nicht etabliert, sodass in diesen Fällen weiterhin eine ergänzende Beurteilung am Lichtmikroskop erforderlich sein kann.

Eine regelmäßige Qualitätssicherung sowie die Kalibrierung von Scannern und Monitoren sind daher unerlässlich. Darüber hinaus kann die digitale Befundung initial mit einer veränderten Befunddauer einhergehen, bis eine ausreichende Routine im Umgang mit den Systemen erreicht ist.^4^, ^6^ Auch beim Einsatz KI‐basierter Systeme bestehen Limitationen. Diese betreffen insbesondere die Abhängigkeit von Trainingsdaten, die begrenzte Generalisierbarkeit sowie regulatorische Anforderungen. KI‐Anwendungen sind derzeit als unterstützende Werkzeuge konzipiert und ersetzen nicht die ärztliche Befundung.^6^, ^9^ Insgesamt erfordert die Nutzung digitaler Pathologie eine sorgfältige Abwägung von Nutzen und Limitationen sowie eine strukturierte Implementierung unter Berücksichtigung technischer und organisatorischer Rahmenbedingungen.

Die digitale Pathologie bietet Chancen für Vernetzung, Standardisierung und Lehre, erfordert jedoch eine sorgfältige technische und organisatorische Implementierung.

### Abgrenzung: Digitale Pathologie und Telepathologie

Die Begriffe digitale Pathologie und Telepathologie werden im klinischen Alltag teilweise synonym verwendet, beschreiben jedoch unterschiedliche Konzepte. Unter digitaler Pathologie wird die vollständige Digitalisierung histologischer Präparate mittels *Whole‐Slide‐Imaging* verstanden, die eine primär digitale Befundung am Bildschirm ermöglicht. Die Telepathologie hingegen bezeichnet die ortsübergreifende Übertragung pathologischer Bildinformationen mit dem Ziel der Fernbefundung oder Konsultation.^1^ Telepathologische Anwendungen können sowohl auf statischen Einzelbildern als auch auf *Live*‐Mikroskopie oder digitalen Ganzobjektträgern basieren. Die digitale Pathologie stellt dabei die technische Grundlage für moderne telepathologische Konzepte dar, da sie eine standardisierte, hochauflösende und reproduzierbare Bildübertragung ermöglicht. In der dermatopathologischen Praxis sind beide Ansätze insbesondere für Konsile und Zweitmeinungen relevant, unterscheiden sich jedoch hinsichtlich technischer Anforderungen, Bildqualität und diagnostischer Aussagekraft.^6^, ^7^

### Dermatopathologische Besonderheiten der digitalen Befundung

Die Anwendung digitaler Pathologie in der Dermatopathologie ist mit spezifischen Anforderungen verbunden. Die Beurteilung melanozytärer Läsionen erfordert eine exakte Darstellung architektonischer und zytologischer Details über mehrere Schnittebenen hinweg. Digitale Systeme ermöglichen die parallele Betrachtung serieller Schnitte und immunhistochemischer Zusatzfärbungen, setzen jedoch eine hohe Bildqualität und konsistente Färbeprotokolle voraus.^4^ Pigment, Schnittartefakte oder Fokussierungsprobleme können die digitale Beurteilbarkeit einzelner Areale beeinträchtigen und erfordern eine kritische Bewertung der Scanqualität. Insbesondere bei stark pigmentierten Läsionen oder sehr feinen epidermalen Veränderungen ist eine sorgfältige technische Qualitätssicherung essenziell.^1^, ^5^ Darüber hinaus gewinnt die digitale Archivierung serieller Schnitte in der Dermatopathologie an Bedeutung, da sie Verlaufskontrollen und Vergleichsbefunde erleichtert. Diese Aspekte unterstreichen, dass die digitale Pathologie in der Dermatopathologie besondere morphologische und technische Anforderungen erfüllen muss, um eine sichere diagnostische Anwendung zu gewährleisten.^2^

### Kognitive Aspekte der digitalen Befundung

Die digitale Befundung histologischer Präparate erfolgt zunehmend in virtuellen Arbeitsumgebungen und ist häufig mit hohen Fallzahlen und längeren Bildschirmarbeitszeiten verbunden. Diese Bedingungen können die kognitive Belastung erhöhen und das Risiko von Ermüdungseffekten beeinflussen. Insbesondere das Konzept der *Decision Fatigue* beschreibt eine nachlassende Entscheidungsqualität bei fortschreitender Befunddauer und steigender Fallzahl, was die diagnostische Verwundbarkeit im Routinebetrieb erhöhen kann.^10^ Neben der reinen Entscheidungserschöpfung gewinnt das Konzept der *cognitive robustness* in der Dermatopathologie an Bedeutung. Es beschreibt die Fähigkeit, auch unter Bedingungen hoher Arbeitsdichte, Wiederholung und digitaler Reizüberflutung eine stabile diagnostische Qualität aufrechtzuerhalten. Digitale Arbeitsumgebungen können diese Robustheit sowohl unterstützen als auch beeinträchtigen, abhängig von Workflow‐Gestaltung, Pausenmanagement und individueller Erfahrung.^11^ Vor diesem Hintergrund ist die digitale Pathologie nicht ausschließlich als technische Transformation zu verstehen, sondern auch als Veränderung kognitiver Arbeitsbedingungen. Aspekte wie Befundrhythmus, ergonomische Gestaltung digitaler Arbeitsplätze und bewusste Strategien zur Reduktion kognitiver Ermüdung spielen eine relevante Rolle für die diagnostische Qualität, insbesondere bei der Befundung hoher Routinemengen am Bildschirm.^10^, ^11^ Hohe Fallzahlen, rechtliche Unsicherheit und der Anspruch maximaler Absicherung können dabei defensive Entscheidungsstrategien begünstigen und die kognitive Belastung im diagnostischen Alltag zusätzlich erhöhen.^12^ Erfahrungen aus der digitalen dermatopathologischen Ausbildung zeigen, dass virtuelle Formate zwar eine hohe Akzeptanz finden, gleichzeitig, aber auch neue Belastungsfaktoren mit sich bringen, die bei der Gestaltung digitaler Arbeits‐ und Befundungsumgebungen berücksichtigt werden sollten.^13^ Studien zur Einführung digitaler Pathologiesysteme zeigen, dass der Übergang von der Glasbefundung zur digitalen Befundung von vielen Pathologen und Dermatopathologen als anspruchsvoll empfunden wird. Neben technischen Aspekten spielen insbesondere individuelle Lernkurven, veränderte Wahrnehmungs‐ und Arbeitsweisen sowie Fragen der Akzeptanz eine zentrale Rolle. Mehrere Arbeiten beschreiben, dass Vertrauen in die digitale Befundung häufig erst im Verlauf strukturierter Einführungs‐ und Validierungsprozesse entsteht.^1^, ^4^

Die digitale Routinebefundung am Bildschirm kann mit erhöhter kognitiver Belastung einhergehen; *Decision Fatigue* und die individuelle kognitive Robustheit beeinflussen dabei die diagnostische Qualität wesentlich.

## ZUKUNFTSPERSPEKTIVEN UND AUSBLICK

Die digitale Pathologie befindet sich in einem dynamischen Entwicklungsprozess, der in den kommenden Jahren voraussichtlich zu einer weiteren Integration in die dermatologische Routinediagnostik führen wird. Technische Fortschritte in der Scannertechnologie, der IT‐Infrastruktur und der Bildverarbeitung tragen dazu bei, digitale Systeme leistungsfähiger und breiter einsetzbar zu machen.

Ein zentrales Entwicklungsfeld ist die zunehmende Etablierung KI‐basierter Assistenzsysteme. Zukünftig ist zu erwarten, dass solche Anwendungen verstärkt zur standardisierten Quantifizierung histologischer Parameter eingesetzt werden, beispielsweise bei der Bewertung von Proliferationsmarkern oder der objektiven Erfassung morphologischer Muster. Voraussetzung hierfür bleibt eine sorgfältige klinische Validierung sowie die transparente Darstellung von Leistungsgrenzen und Anwendungsbereichen. Der Einsatz dieser Systeme wird weiterhin ergänzend zur ärztlichen Befundung erfolgen. Systematische Übersichten zeigen, dass KI‐basierte Klassifikationsmodelle in der Hautkrebsdiagnostik in experimentellen Settings eine mitunter gleichwertige oder überlegene Performance im Vergleich zu menschlichen Experten erreichen können. Gleichzeitig weisen diese Arbeiten darauf hin, dass viele Studien unter stark vereinfachten Bedingungen durchgeführt wurden und die Übertragbarkeit auf den klinischen Alltag, insbesondere bei histopathologischen Ganzschnittpräparaten, limitiert bleibt.^14^

Ein weiterer Schwerpunkt liegt in der verbesserten Vernetzung zwischen dermatologischen Praxen, Kliniken und pathologischen Instituten. Digitale Plattformen ermöglichen einen strukturierten Austausch histologischer Befunde und fördern interdisziplinäre Konsile. Insbesondere in Regionen mit begrenzter Verfügbarkeit spezialisierter dermatopathologischer Expertise kann dies zur Qualitätssicherung beitragen.^5^, ^7^

Darüber hinaus eröffnet die digitale Pathologie Perspektiven für die Kombination histologischer Bilddaten mit klinischen, molekularen und genetischen Informationen. Solche multimodalen Ansätze könnten zukünftig zu einer präziseren Klassifikation dermatologischer Erkrankungen beitragen. Derzeit befinden sich entsprechende Konzepte überwiegend im Forschungsstadium und erfordern standardisierte Datenformate sowie interoperable Systeme. Auch in der Aus‐ und Weiterbildung ist mit einer weiteren Bedeutung digitaler Pathologiesysteme zu rechnen. Digitale Lehrarchive und virtuelle Fallkonferenzen ermöglichen einen ortsunabhängigen Zugang zu dermatopathologischen Inhalten und unterstützen eine standardisierte Wissensvermittlung.^6^, ^13^

Zusammenfassend lässt sich festhalten, dass die digitale Pathologie langfristig das Potenzial besitzt, diagnostische Prozesse in der Dermatologie zu unterstützen und zu strukturieren. Eine erfolgreiche Weiterentwicklung setzt jedoch voraus, dass technische Innovationen mit klaren Qualitätsstandards, validierten Anwendungen und definierten Verantwortlichkeiten einhergehen.

## DANKSAGUNG

Open access Veröffentlichung ermöglicht und organisiert durch Projekt DEAL.

## INTERESSENKONFLIKT

Keiner.

## [CME Questions – Lernerfolgskontrolle]

1. Was bezeichnet der Begriff *digitale Pathologie* korrekt?
  - Die Speicherung ausgewählter histologischer Einzelbilder.
  - Die Fernbefundung histologischer Präparate per Videomikroskopie.
  - Die vollständige Digitalisierung histologischer Präparate zur primär digitalen Befundung.
  - Die digitale Archivierung histologischer Befunde ohne Nutzung digitaler Präparate.
  - Die automatisierte histologische Diagnose ohne ärztliche Beteiligung.
2. Welche Technologie bildet die technische Grundlage der digitalen Pathologie?
  - Fluoreszenzmikroskopie.
  - Telekonferenzsysteme.
  - *Whole‐Slide‐Imaging*.
  - Molekulargenetische Analyse.
  - Virtuelle Realität.
3. Welche Voraussetzung ist für eine diagnostisch sichere digitale Befundung besonders wichtig?
  - Verwendung ausschließlich verlustfreier Bildformate.
  - Konstante Bildqualität und kalibrierte Monitore.
  - Hohe Internetgeschwindigkeit allein.
  - Vollständige Automatisierung der Befundung.
  - Verzicht auf Glasobjektträger.
4. Welcher Ansatz hat sich bei der Einführung digitaler Pathologie in der Routine bewährt?
  - Sofortige vollständige Umstellung ohne Übergangsphase.
  - Hybrider Workflow mit paralleler Glas‐ und Digitalbefundung.
  - Ausschließliche Nutzung digitaler Präparate für Konsile.
  - Einführung nur für immunhistochemische Färbungen.
  - Nutzung ausschließlich zu Lehrzwecken.
5. Was ist ein zentrales Ziel der Validierung digitaler Pathologiesysteme?
  - Verkürzung der Befundzeit.
  - Sicherstellung der diagnostischen Gleichwertigkeit zur Glasbefundung.
  - Reduktion der Speicheranforderungen.
  - Erhöhung der Automatisierungsrate.
  - Vereinfachung der Abrechnung.
6. In welchem Bereich bietet die digitale Pathologie in der Dermatologie einen besonderen Nutzen?
  - Ausschließlich bei infektiösen Dermatosen.
  - Bei Konsilen, Verlaufskontrollen und standardisierter Dokumentation.
  - Nur bei seltenen genetischen Erkrankungen.
  - Nur in der Forschung.
  - Ausschließlich bei intraoperativen Schnellschnitten.
7. Welche besondere Anforderung besteht bei der digitalen Beurteilung melanozytärer Läsionen?
  - Verzicht auf serielle Schnitte.
  - Verwendung geringerer Auflösung.
  - Hohe Bildqualität und sorgfältige Qualitätssicherung.
  - Ausschluss immunhistochemischer Färbungen.
  - Einsatz ausschließlich automatischer KI‐Systeme.
8. Welche Aussage zum Einsatz künstlicher Intelligenz in der digitalen Pathologie trifft zu?
  - KI ersetzt die ärztliche Befundung.
  - KI darf ohne klinische Validierung eingesetzt werden.
  - KI dient der unterstützenden Analyse unter ärztlicher Kontrolle.
  - KI ist nur für Forschungszwecke zulässig.
  - KI liefert auch bei niedriger Bildqualität zuverlässige Diagnosen.
9. Welche Limitation ist mit der digitalen Pathologie verbunden?
  - Fehlende Einsatzmöglichkeiten in der Dermatologie.
  - Hohe Anforderungen an IT‐Infrastruktur und Datenspeicherung.
  - Grundsätzlich unzureichende diagnostische Aussagekraft.
  - Ausschluss der Nutzung für Lehre.
  - Unmöglichkeit der Archivierung.
10. Welche Aussage zu rechtlichen und abrechnungstechnischen Aspekten ist korrekt?
  - Diese sind vollständig geklärt und einheitlich geregelt.
  - Die Haftung geht bei digitaler Befundung auf den Softwarehersteller über.
  - Digitale Pathologie ist in allen Bereichen gesondert abrechenbar.
  - Rechtliche, haftungsrechtliche und abrechnungstechnische Rahmenbedingungen sind derzeit nicht vollständig geklärt.
  - Digitale Pathologie ist unabhängig von wirtschaftlichen und abrechnungstechnischen Aspekten implementierbar.

Liebe Leserinnen und Leser, der Einsendeschluss an die DDA für diese Ausgabe ist der 30. Oktober 2026.

Die richtige Lösung zum Thema Komplikationen von Laser und Energie‐basierten Systemen in der Dermatologie: Klassifikation, Management und Prävention in Heft 3/2026 ist: 1b, 2c, 3c, 4b, 5b, 6b, 7a, 8d, 9d, 10a

Bitte verwenden Sie für Ihre Einsendung das aktuelle Formblatt auf der folgenden Seite oder aber geben Sie Ihre Lösung online unter http://jddg.akademie-dda.de ein.
